# Effect of denosumab switched from bisphosphonates in preventing joint destruction in postmenopausal rheumatoid arthritis patients with anti-cyclic citrullinated peptide antibodies

**DOI:** 10.1186/s13018-021-02271-2

**Published:** 2021-02-04

**Authors:** Yu Mori, Takuya Izumiyama, Hiroaki Kurishima, Masayuki Kamimura, Kazuyoshi Baba, Naoko Mori, Eiji Itoi

**Affiliations:** 1 Department of Orthopedic Surgery, Tohoku University Graduate School of Medicine, 1-1 Seiryo machi, Aobaku, Sendai, Miyagi 980-8574 Japan; 2 Department of Diagnostic Radiology, Tohoku University Graduate School of Medicine, 1-1 Seiryo machi, Aobaku, Sendai, Miyagi 980-8574 Japan

**Keywords:** Denosumab, Rheumatoid arthritis, Anti-cyclic citrullinated peptide antibody, Bone erosion, Joint destruction, Modified total Sharp score

## Abstract

**Introduction:**

This study aimed to determine the effects of denosumab treatment on the joint destruction of Japanese females with rheumatoid arthritis (RA) and anti-cyclic citrullinated peptide (CCP) antibodies.

**Materials and methods:**

This retrospective longitudinal study included 56 patients treated with denosumab and 50 patients treated with bisphosphonate. All participants were positive for anti-CCP antibodies. All patients also had a history of osteoporosis treatment with bisphosphonate, which was either continued or switched to 60 mg of subcutaneous denosumab injection every 6 months. To assess the progression of joint destruction, hand and foot radiographs were taken, and changes in modified total Sharp score (mTSS), erosion score (ERO), and joint space narrowing score (JSN) were evaluated at 12 months and 24 months. The changes in BMD of the lumbar spine and hip were also assessed at 12 months.

**Results:**

At 12 months, there were significant differences in the change of ERO (*p* = 0.015) and mTSS (*p* = 0.01). Similarly, there were significant differences in the change of ERO (*p* = 0.013) and mTSS (*p* = 0.003) at 24 months. In contrast, no significant difference was observed in the changes of JSN and clinical parameters. There were significant differences in the changes in BMD in the femoral neck (*p* = 0.011) and total hip (*p* = 0.012).

**Conclusion:**

Denosumab treatment might be effective for the inhibition of bone erosion progression in the patients with RA, and it potentially contributes to the treatment of osteoporosis and prevention of destructive arthritis in patients with switching treatment from bisphosphonate**.**

## Introduction

Rheumatoid arthritis (RA) is an autoimmune disease characterized by inflammatory synovitis and bone and cartilage destruction [1, 2]. Patients with RA also carry a risk of general bone loss and osteoporosis [3–5]. Destructive arthritis and general bone loss are considered to be related to activated osteoclasts. The receptor activator of nuclear factor kappa B (RANK) ligand (RANKL) and its related signals are essential for osteoclast development, activation and survival [1, 2, 6–8]. Proinflammatory cytokines, including tumor necrosis factor-alpha (TNF-α), interleukin (IL)-1, IL-6, and IL-17 are related with the induction of RANKL in inflammatory cells, such as T lymphocytes and synovial cells [9, 10]. Bone resorption and destruction by activated RANKL signaling are associated with the progression of joint deformation with active synovitis. In contrast, the progression of bone erosion and destruction are observed in patients without marked synovial inflammation [11]. Thus, RANKL pathway seems to be activated in patients with RA regardless of inactivated synovitis.

Denosumab is a fully human monoclonal IgG2 antibody, which acts by binding RANKL and suppressing the activity of the RANKL signaling pathway. The suppression of RANKL by denosumab inhibits the bone resorption of osteoclasts and may prevent bone erosion and joint deformity in an experimental arthritis model [12]. Randomized control studies and systematic reviews demonstrated the therapeutic effects of denosumab for the prevention of osteoporosis and osteoporotic fractures in postmenopausal patients [13–16]. In contrast, a few randomized control studies and several small-scale clinical studies were performed for the assessment of the therapeutic effects of the denosumab for the prevention of bone erosion in the patients with RA [11, 17–20]. The results of these studies demonstrated the inhibition of bone erosion progression compared with placebo. These studies were conducted among patients with conventional synthetic disease-modifying antirheumatic drugs (csDMARDs), including methotrexate. On the other hand, the patients with RA with biological DMARDs (bDMARDs) were not included in these studies, and the effect of denosumab in the prevention of the bone erosion in the patients with RA treated with bDMARDs still remains unclear. A previous study reported that denosumab had significantly reduced bone erosion in patients with anti-cyclic citrullinated peptide (anti-CCP), which is a risk factor for radiographic damage [21]. However, no other studies have demonstrated the relation between anti-CCP antibody and the prevention effect of denosumab against bone erosion. The therapeutic effects of denosumab for bone erosion among patients with a positive anti-CCP antibody status have not been determined. In addition, previous studies reported the effect of denosumab in the prevention of bone erosion for only 12 months; no studies have reported the long-term effects of denosumab yet. The continuity of the denosumab for inhibition of bone destruction was also undetermined.

The main aim of the present study was to evaluate the effect of denosumab treatment on joint destruction inhibition in patients with anti-CCP antibodies. In the clinical setting, refractory patients with RA have been treated with bDMARDs for synovial inflammation and inhibition of joint destruction. This study included patients with RA treated with bDMARDs and tested the inhibition of bone erosion in participants with bDMARDs treatment to clarify the efficacy of denosumab for the prevention of bone erosion, even in the presence of bDMARD treatment. We also evaluated the effect of denosumab on the improvement of BMD and prevention of osteoporotic fractures.

## Materials and methods

### Patients

This retrospective longitudinal study was conducted in accordance with the ethical standards of the Declaration of Helsinki and approved by the Institutional Review Board of our institute (Approval number: 2020-1-814). Informed consent was obtained from all patients before participation in this study. The study population included 106 Japanese postmenopausal female patients with RA and osteoporosis, according to the American College of Rheumatology (ACR) classification criteria (1987) or ACR/European League against Rheumatism criteria [22, 23]. All patients were positive for anti-CCP antibodies, and also fulfilled the Japanese society of Osteoporosis criteria for the diagnosis of glucocorticoid-induced osteoporosis or primary osteoporosis [24, 25]. Patients were enrolled at our institution from April 2015 through March 2020. All patients had a history of bisphosphonate treatment; they either continued bisphosphonate treatment or switched to subcutaneous injection of 60 mg of denosumab every 6 months. The reasons for switching from bisphosphonates to denosumab were patient preference for convenience and inadequate improvement in bone mineral density by bisphosphonates. In addition, all patients were treated with an active form of Vitamin D. Then, 0.75 μg of eldecalcitol or 1 μg of alphacalcidol were administered before the enrollment of the study. We excluded patients with parathyroid disease, chronic severe renal dysfunction, hypocalcemia, and malabsorption disease, as well as patients who had been treated with parathyroid hormone and romosozumab. Furthermore, we excluded patients with avascular necrosis of the hip and vertebral fractures caused by high-velocity injuries.

### Clinical variables

Baseline general characteristics were recorded in detail, including age, height, bodyweight, and body mass index (BMI) in patients with RA at the start of the study. We measured the RA disease activity by calculating composite disease activity scores (DAS). C-reactive protein based DAS28 (DAS28-CRP) includes the number of swollen and tender joints (out of a total of 28), a global visual analog scale (VAS) score, and the C-reactive protein level. The titer of anti-CCP antibody and matrix metalloproteinase-3 (MMP3) were also assessed. The Health Assessment Questionnaire (HAQ) was administered to assess functional disability. All clinical assessments were performed at baseline and at 12 months of follow-up. The duration of disease and treatment of rheumatoid arthritis were also recorded.

### Radiographic assessment of the modified Sharp score

Hand and foot radiographs were taken at baseline, 12 months and 24 months. For the assessment of the modified total sharp score (mTSS), two rheumatologists independently assessed the radiographs using the modified Sharp/van der Heijde method with blinded clinical information of patients, and the average score was calculated and used in the analysis [11, 26]. Changes in the modified Sharp erosion score (ERO), modified Sharp joint space narrowing score (JSN), and mTSS were evaluated at 12 and 24 months. The structural remission rate at 12M (the change of mTSS ≤ 0.5) was calculated in bisphosphonates and denosumab treatment groups [23].

### Bone mineral density measurement and vertebral fracture assessment

Bone mineral density (BMD) measurement and vertebral fracture assessment were performed as previously described in the literature [27]. We measured BMD (g/cm^2^) at the AP lumbar spine (vertebrae L2–4) and left hip (total hip and femoral neck) by using dual-energy X-ray absorptiometry (Discovery DXA system; Hologic, Waltham, MA, USA). All procedures were performed according to the manufacturer’s standardized protocols at baseline and 12 months. All BMD results were expressed as absolute values (g/cm^2^). Thoracolumbar radiographs were obtained in all patients to detect vertebral fractures, including both painful vertebral fractures and asymptomatic morphologic vertebral fractures at baseline and 12 months.

### Statistical analysis

All results were expressed as the mean ± standard deviation. At baseline, 12 months and 24 months, comparisons of clinical parameters between denosumab and bisphosphonate treatment groups were performed using the Mann–Whitney *U* test. This test was also performed to identify the differences in the change in BMD of the lumbar spine, femoral neck, and total hip between two groups at 12 months. Similarly, the changes of mTSS, ERO, and JSN were compared using Mann–Whitney *U* test between the groups at 12 months and 24 months. The structural remission rate at 12M (the change of mTSS ≤ 0.5) was compared using Fisher's exact test. The usage of bDMARDs, bisphosphonates, and active form of vitamin D were compared between two groups using Fisher’s exact test. All statistical tests were two-sided, with a *p* value of less than 0.05 considered statistically significant. All analyses were performed using JMP version 15 (SAS, Cary, NC, USA).

## Results

In this study, sixteen patients with RA using bisphosphonate and 12 patients with RA using denosumab were not included in the study because they were negative for anti-CCP antibodies. Finally, 106 postmenopausal Japanese women with rheumatoid arthritis were enrolled. There were 50 and 56 patients in the bisphosphonate-continuing group and the denosumab group, respectively. There were no apparent side effects of bisphosphonates and denosumab during the treatment, including hypocalcemia and interstitial pneumonia. Table 1 shows the clinical characteristics of these two groups. Significant intergroup differences were observed related to age [67.6 (7.4) vs. 71.1 (8.2), *p* = 0.035] and usage of methotrexate [38 (76%) vs. 26 (46.4), *p* = 0.02]. There were no significant differences in body weight, BMI, and usage of prednisone and bDMARDs, including etanercept, golimumab, tocilizumab, sarilumab, and abatacept. In the assessment of disease activity of RA, such as DA28-CRP and the titer of anti-CCP antibody, and joint deformity including ERO, JSN, and mTSS, there were no significant differences between the two groups. Similarly, there were no significant differences between the groups in the comparison of BMD and previous vertebral fractures. The usage of bisphosphonates and active form of vitamin D were shown in Table 5 in Appendix. There were no significant differences in the usage of bisphosphonates and vitamin D between two groups.

**Table 1:** Comparison of clinical measurements in the patients with RA treated with bisphosphonates and denosumab at enrollment

Variable	Bisphosphonate group (*n* = 50)	Denosumab group (*n* = 56)	*p* value
Age (years)	67.6 (7.4)	71.1 (8.2)	0.035*
Height (cm)	152.2 (6.4)	151.4 (5.7)	0.57
Body weight (kg)	54.3 (10.8)	52.3 (10.1)	0.32
BMI	23.4 (4.4)	22.8 (3.9)	0.73
Disease duration (years)	9.7 (5.1)	11.3 (5.2)	0.12
bDMARDs use, *n* (%)	30 (60)	25 (44.6)	0.12
Etanercept (50 mg), *n* (%)	2 (4)	3 (5.4)	0.74
Golimumab (50 mg), *n* (%)	4 (8)	3 (5.4)	0.58
Tocilizumab (162 mg), *n* (%)	8 (16)	7 (12.5)	0.6
Sarilumab (200mg), *n* (%)	4 (8)	4 (7.1)	0.86
Abatacept (125mg), *n* (%)	12 (24)	8 (14.3)	0.2
MTX use, *n* (%)	38 (76)	26 (46.4)	0.02*
MTX dose, mg/week	7.3 (1.8)	6.5 (2.1)	0.11
Prednisone use *n* (%)	22 (44)	18 (32.1)	0.21
Prednisone dose, mg/day	5.36 (1.4)	5.4 (2.3)	0.72
Modified sharp erosion score (0–280)	36.1 (43.1)	29.6 (33.9)	0.63
Modified Sharp JSN score (0-168)	24.1 (36.5)	23.1 (29.3)	0.21
Modified total sharp score (0–448)	60.1 (79.3)	52.5 (62.5)	0.98
DAS28-CRP	3.1 (1.1)	2.83 (1.2)	0.28
VAS	28.8 (17.7)	29.2 (19.7)	0.82
mHAQ	0.52 (0.43)	0.64 (0.77)	0.87
ACPA titer (U/ml)	109.7 (171.3)	141.1 (246.9)	0.56
MMP3 (ng/ml)	94.3 (48.3)	104.9 (69.9)	0.74
BMD			
Lumbar spine (g/cm^2^)	0.89 (0.17)	0.86 (0.16)	0.29
Femoral neck (g/cm^2^)	0.58 (0.11)	0.56 (0.11)	0.52
Total hip (g/cm^2^)	0.69 (0.13)	0.67 (0.14)	0.44
Vertebral fracture, *n* (%)	10 (20)	14 (25)	0.45

Results are expressed as the mean and standard deviation

*BMI* body mass index, *DMARDs* disease-modifying antirheumatic drugs, *MTX* methotrexate, *DAS* disease activity score, *CRP* C-reactive protein, *VAS* visual analogue scale, *HAQ* Health Assessment Questionnaire, *ACPA* anti-cyclic citrullinated peptide antibody, *MMP3* matrix metalloproteinase, *BMD* bone mineral density

*p < 0.05 by Mann–Whitney *U* test

**Fig. 1:**
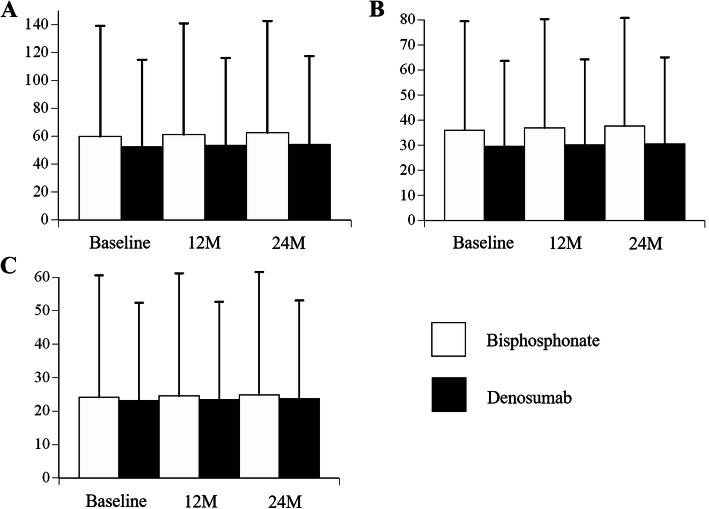
Comparison of the modified Sharp scores at baseline, 12 months, and 24 months. **a** Modified total Sharp score (0–448). **b** Modified Sharp erosion score (0–280). **c** Modified Sharp joint space narrowing score (0–168). Results are expressed as the mean and standard deviation

**Fig. 2:**
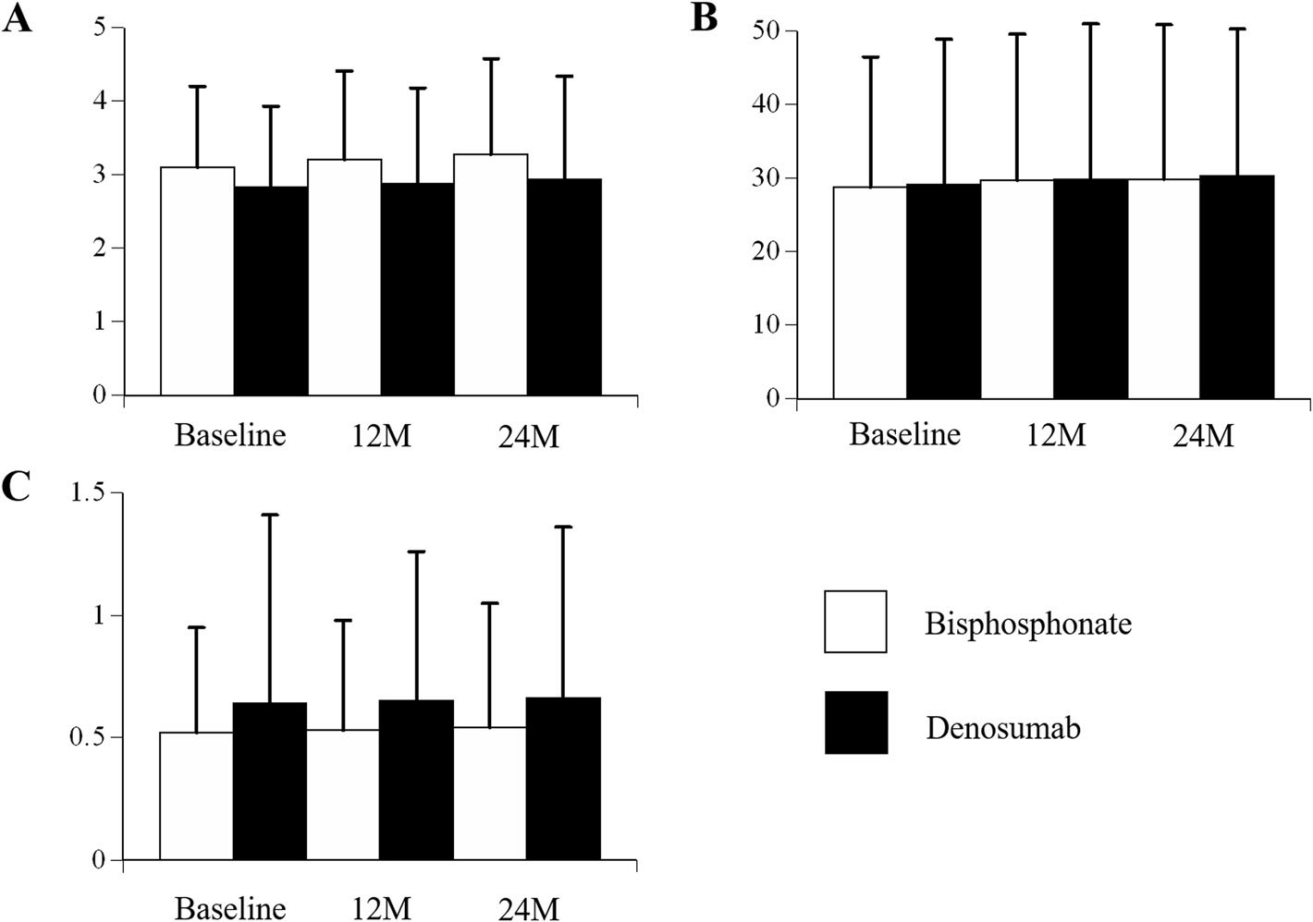
Comparison of the clinical scores at baseline, 12 months, and 24 months. **a** DAS28-CRP. **b** VAS. **c** mHAQ. Results are expressed as the mean and standard deviation. *DAS* disease activity score, *CRP* C-reactive protein, *VAS* visual analogue scale, *HAQ* Health Assessment Questionnaire

The scores of mTSS, JSN, and ERO at baseline, 12 months, and 24 months are shown in Fig. 1, respectively. There were no significant differences between the two groups. Figure 2 shows clinical scores of DAS28-CRP, VAS, and mHAQ at baseline, 12 months, and 24 months, respectively. There were no significant differences between the groups.

**Fig. 3:**
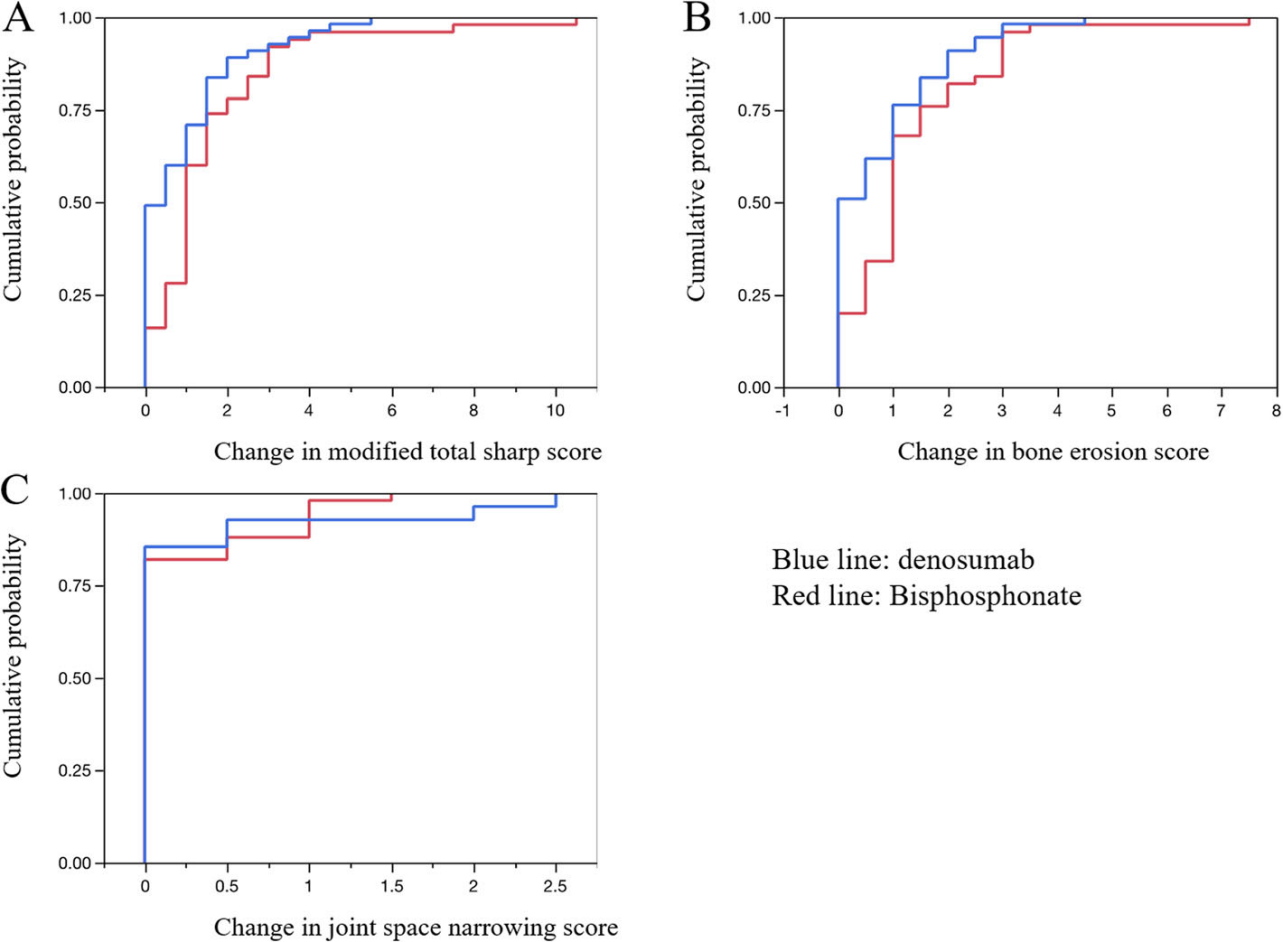
Cumulative probability plots of the changes from baseline at 12 months. **a** Modified total Sharp score. **b** Modified Sharp erosion score. **c** Modified Sharp joint space narrowing score

Table 2 compares of the changes of ERO, JSN, mTSS scores, and clinical scores at 12 months. There were significant differences in the change of ERO [1.16 (1.47) vs. 0.72 (1.58), *p* = 0.015] and mTSS [1.56 (2.15) vs. 1.12 (2.31), *p* = 0.01]. Cumulative probability blots for the changes in ERO, JSN, and mTSS scores at 12 months are shown in Fig. 3. A higher structural remission rate (the change of mTSS ≤ 0.5) was observed in the denosumab treatment group (28 vs. 58.9%; *p* < 0.001). In contrast, no significant difference was observed in the change of JSN and clinical parameters, including DAS28-CRP, VAS and mHAQ.

**Table 2:** Comparison of the changes of ERO, JSN, mTSS scores, and clinical scores at 12 months

Variable	Bisphosphonate group (*n* = 50)	Denosumab group (*n* = 56)	*p* value
Modified sharp erosion score	1.16 (1.47)	0.72 (1.58)	0.015*
Modified sharp JSN score	0.24 (0.55)	0.27 (0.84)	0.92
Modified total sharp score	1.56 (2.15)	1.12(2.31)	0.01**
DAS28-CRP	0.1 (0.74)	0.04 (0.53)	0.9
VAS	0.96 (18.1)	0.82 (15.7)	0.82
mHAQ	0.014 (0.23)	0.021 (0.31)	0.72

Results are expressed as the mean and standard deviation

*DAS* disease activity score, *CRP* C-reactive protein, *VAS* visual analogue scale, *HAQ* Health Assessment Questionnaire

*p < 0.05; **p < 0.01 by Mann–Whitney *U* test

**Fig. 4:**
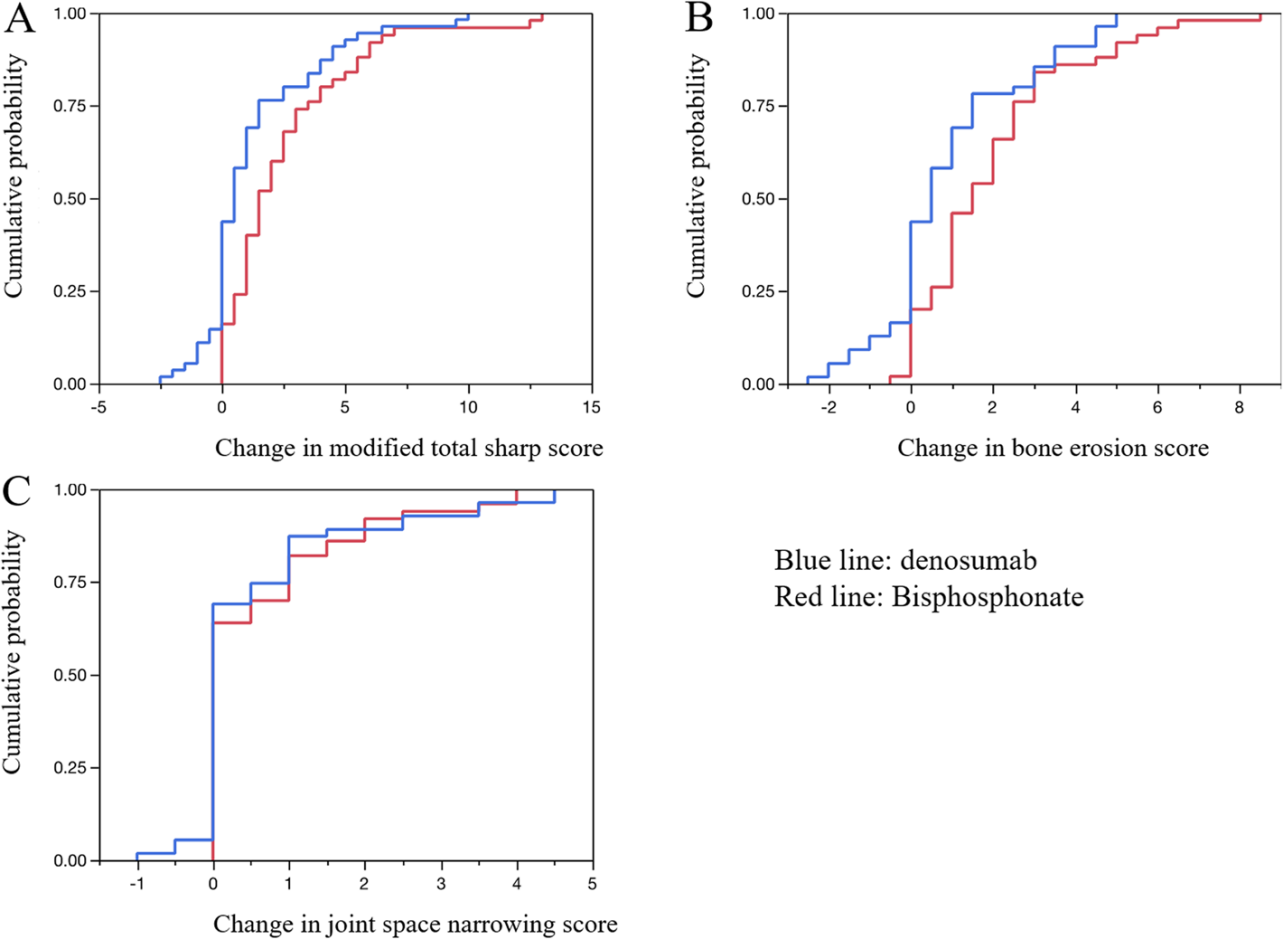
Cumulative probability plots of the changes from baseline at 24 months. **a** Modified total Sharp score. **b** Modified Sharp erosion score. **c** Modified Sharp joint space narrowing score

Table 3 demonstrates the comparison of the changes of ERO, JSN and mTSS at 24 months. There were significant differences in the change of ERO [1.56 (1.62) vs. 1.07 (2.38), *p* = 0.013] and mTSS [2.52 (2.97) vs. 1.73 (4.36), *p* = 0.003]. Cumulative probability blots for the changes in ERO, JSN, and mTSS scores at 24 months are shown in Fig. 4. Similar to the assessment at 12 months, no significant differences were observed in the change of JSN and clinical parameters, including DAS28-CRP, VAS, and mHAQ.

**Table 3:** Comparison of the changes of ERO, JSN, mTSS scores, and clinical scores at 24 months

Variable	Bisphosphonate group (*n* = 50)	Denosumab group (*n* = 56)	*p* value
Modified sharp erosion score	1.56 (1.62)	1.07 (2.38)	0.013*
Modified sharp JSN score	0.56 (1.1)	0.61 (1.37)	0.67
Modified total sharp score	2.52 (2.97)	1.73 (4.36)	0.003**
DAS28-CRP	0.18 (0.96)	0.11 (0.72)	0.84
VAS	1.26 (20.5)	0.98 (17.6)	0.87
mHAQ	0.019 (0.27)	0.026 (0.32)	0.78

Results are expressed as the mean and standard deviation

*DAS* disease activity score, *CRP* C-reactive protein, *VAS* visual analogue scale, *HAQ* Health Assessment Questionnaire

*p < 0.05; **p < 0.01 by Mann–Whitney *U* test

**Fig. 5:**
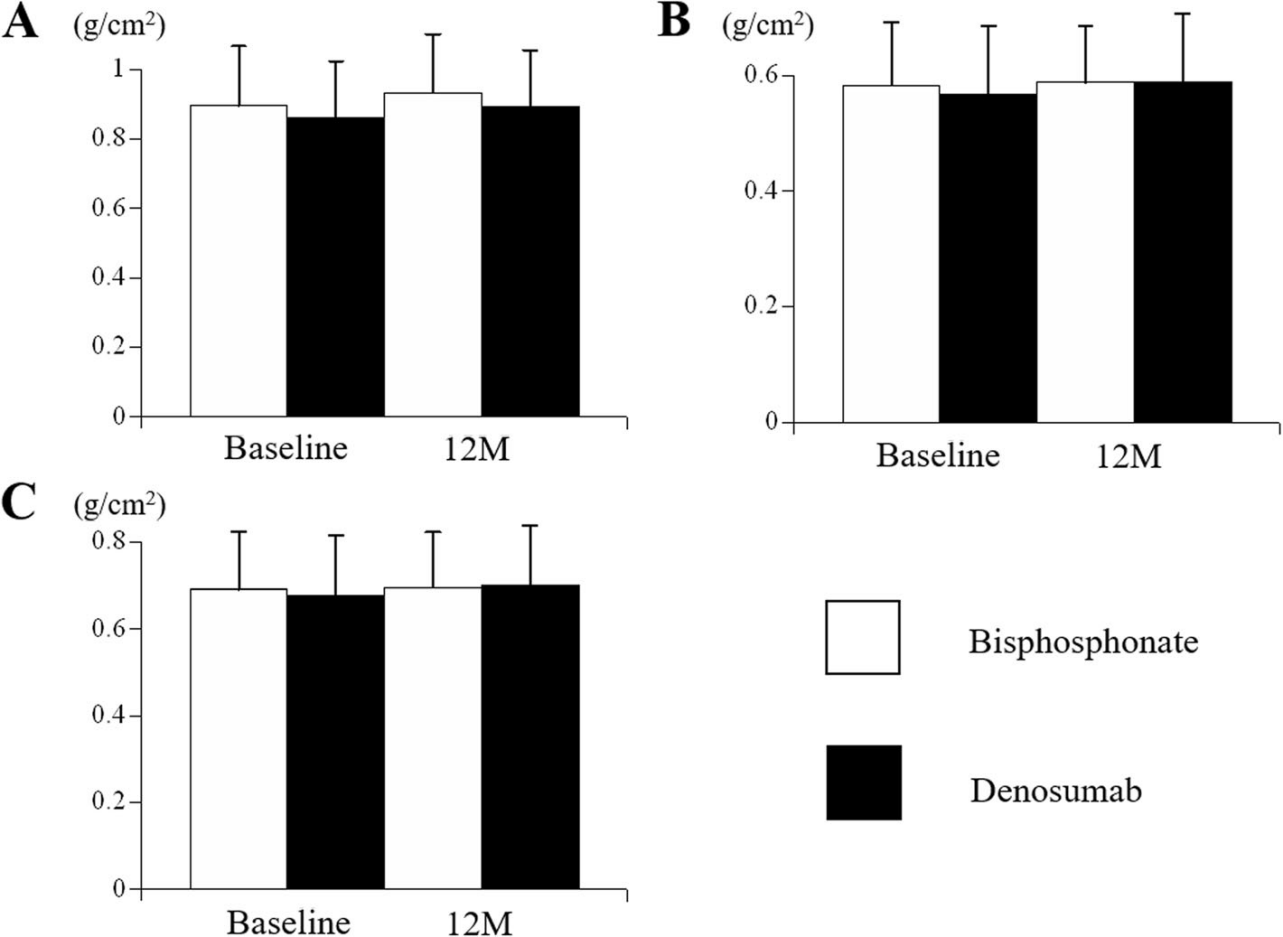
Comparison of bone mineral density at baseline and 12 months. **a** Lumbar spine. **b** Femoral neck. **c** Total hip. Results are expressed as the mean and standard deviation

The BMD of lumbar spine, femoral neck, and total hip at baseline and 12 months are shown in Fig. 5. There were no significant differences between the two groups.

The changes of BMD were shown in Table 4. There were significant differences related to the BMD changes of femoral neck [0.0045 (0.033) vs. 0.021 (0.042), *p* = 0.011] and total hip [0.0042 (0.056) vs. 0.027 (0.062), *p* = 0.012]. In contrast, there were no significant differences in the BMD of lumbar spine and the incidence of vertebral fractures.

**Table 4:** Comparison of the changes of bone mineral density and radiographic vertebral fractures at 12 months

Variable	Bisphosphonate group (*n* = 50)	Denosumab group (*n* = 56)	*p* value
BMD			
Lumbar spine (g/cm^2^)	0.034 (0.083)	0.033 (0.072)	0.32
Femoral neck (g/cm^2^)	0.0045 (0.033)	0.021 (0.042)	0.011*
Total hip (g/cm^2^)	0.0042 (0.056)	0.027 (0.062)	0.012*
Vertebral fracture, *n* (%)	2 (4)	1 (1.8)	0.49

Results are expressed as the mean and standard deviation

*p < 0.05 by Mann–Whitney *U* test

## Discussion

The present study observed significant differences related to the change of mTSS and ERO between bisphosphonate treatment group and denosumab treatment group in patients with a positive anti-CCP antibody status at 12 and 24 months. In contrast, there was no significant difference in the change of JSN. Similarly, significant differences were observed in the change of BMD of femoral neck and total hip between the groups. However, there was no significant difference in the change of BMD of lumbar spine. These results are consistent with previously conducted large-scale studies [11, 18, 21]. In this study, patients undergoing bDMARDs treatment were enrolled in both groups. Denosumab treatment was effective for the inhibition of bone erosion, even in the presence of bDMARDs. The present study included the patients with switching treatment from bisphosphonate to denosumab and demonstrated the efficacy of the switching treatment for the inhibition of the bone erosion and destruction, like as the improvement of BMD in patients with RA.

Previous studies have also discussed the efficacy of denosumab for the inhibition of bone erosion. The changes in mTSS were remarkably greater in patients with anti-CCP antibodies [21]. The anti-CCP antibody titer has been demonstrated as a predictor of bone erosion and destruction in patients with RA [28, 29]. Patients with anti-CCP antibodies were likely to have the risk of advanced radiographic progression. However, denosumab demonstrated significant inhibition of bone erosion in patients with anti-CCP antibodies [21]. These results were consisted with those of present study. These findings indicted that denosumab is expected to suppress the progression of bone erosion and destruction in patients with anti-CCP antibody positive status.

Previous studies were performed to identify the effect of denosumab treatment on the inhibition of bone erosion and destruction in the patients with RA without treatment history of bisphosphonate and bDMARDs [11, 18, 21]. Osteoporosis treatment guidelines recommend bisphosphonate at first-line treatment for patient with risks of osteoporosis and osteoporotic fractures [24, 25]. Patients with rheumatological disease have the high risks of general bone loss and osteoporotic fractures, and should be treated with bisphosphonate or denosumab [30, 31]. Therefore, patients with RA without a history of bisphosphonate and bDMARD treatment seemed to be uncommon and different from actual clinical settings. In contrast, some studies indicated that the effect of switching treatment from bisphosphonates to denosumab for the improvement of BMD in patients with RA. However, previous studies did not demonstrate the effect of the prevention of bone erosion and destruction by switching bisphosphonates to denosumab [32, 33]. Since the present study included patients treated with bDMARDs and bisphosphonates, it can be assumed that the study was conducted under conditions similar to actual clinical practice. The present study was the first to demonstrate the efficacy of the switching treatment from bisphosphonate to denosumab for the inhibition of bone erosion and destruction.

The mTSS was the sum of ERO and JSN. The significant difference in mTSS and ERO between the two groups was considered to reflect the inhibitory effect of denosumab on bone erosion. On the other hand, there was no significant difference in JSN, suggesting that neither denosumab nor bisphosphonate had an inhibitory effect on cartilage destruction. In this study, the increase in mTSS at 12 months in the bisphosphonate and denosumab groups exceeded 0.5, which did not correspond to structural remission [23]. However, a higher structural remission rate (the change of mTSS ≤ 0.5) was observed in the denosumab treatment group (28 vs. 58.9%; *p* < 0.001). The results suggest that switching denosumab treatment from bisphosphonate may be advantageous for structural remission.

In this study, we compared and observed the inhibitory effect of denosumab on bone erosion and destruction at 12 and 24 months after switching treatment. Previous studies have confirmed the inhibitory effect of denosumab on bone erosion at 12 months [11, 18, 21], but its effects over a longer period of time remain undetermined. To our best knowledge, the present study was the first to demonstrate the inhibitory effect of denosumab on inhibition of bone erosion and destruction over a 24-month period.

The results of the present study demonstrated that the changes in BMD of femoral neck and total hip were significantly higher in denosumab group. In contrast, there was no significant difference in the change of BMD of lumbar spine. The participants in this study fulfilled the criteria of either bisphosphonate continuation group or the denosumab switchover group; no bisphosphonate-naive patients were included. Therefore, we considered the possibility that significant differences in BMD of lumbar spine were not observed between the two groups due to the effect of the prior bisphosphonate use. Furthermore, a previous study reported that degenerative disease of the lumbar spine and previous vertebral fracture increased the BMD of the lumbar spine in elderly patients [34]. Other studies have reported that the BMD of the femur had a stronger association with the risk factors of osteoporosis compared to the BMD of the lumbar spine in patients with rheumatological diseases [5, 35]. The authors considered that lack of a significant difference in the change of lumbar spine BMD may have been due to its susceptibility to age-related degeneration and vertebral fractures.

There are several limitations in the present study. First, the present study was performed as a retrospective study and the number of patients was small. Authors did not perform the adjustment of confounding factors of participants in this study. Further large-scale prospective studies with the adjustment of confounding factors are required to confirm the results in this study. Second, the treatment agents of rheumatoid arthritis and treatment duration were not uniform among the study patients. Therefore, future studies should consider assessing the treatment effect of denosumab for erosion inhibition in patients with uniform RA treatment history. Third, the types of bisphosphonates used prior to the enrollment of this study and the duration of their use were not uniform. The types of bisphosphonates used continuously were also not uniform. Fourth, the measurement of anti-CCP antibody was not performed after denosumab treatment. The effect of denosumab for decreasing the titer of anti-CCP antibody remained unclear. Finally, the serum bone turnover markers and serum vitamin D levels were not measured in the present study.

In conclusion, the present study indicated significant differences related to the change of mTSS and ERO between bisphosphonate and denosumab treatment groups in the patients with a positive anti-CCP antibody status. In contrast, denosumab treatment did not affect the change of JSN. Similarly, denosumab treatment provided greater improvement in the BMD of the femoral neck and total hip. Denosumab treatment was effective for the inhibition of bone erosion, even in the presence of bDMARDs. Denosumab may contribute to both the treatment of osteoporosis and the prevention of the destructive arthritis in patients with RA. The results of present study are exploratory, and the authors consider that further follow-up is needed for causal inference.

## Data Availability

All data generated or analyzed during this study are included in this published article.

## Appendix

**Table 5:** Comparison of the bisphosphonate and active vitamin D treatment

Variable	Bisphosphonate group (*n* = 50)	Denosumab group (*n* = 56)	*p* value
Bisphosphonates			
Alendronate (35 mg), *n* (%)	16 (32)	22 (39.3)	0.43
Risedronate (17.5 mg), *n* (%)	14 (28)	13 (23.2)	0.57
Minodronate (50 mg), *n* (%)	20 (40)	21 (37.5)	0.79
Vitamin D			
Eldecalcitol (0.75 mg), *n* (%)	41 (82)	51 (91.1)	0.17
Alphacalcidol, (1 mg), *n* (%)	9 (18)	5 (8.9)	0.17

## Abbreviations

ACPA: Anti-cyclic citrullinated peptide antibody
ACR: American College of Rheumatology
BMD: Bone mineral density
BMI: Body mass index
CCP: Cyclic citrullinated peptide
CRP: C reactive protein
DAS: Disease activity score
DMARDs: Disease-modifying antirheumatic drugs
ERO: Modified Sharp erosion
HAQ: Health Assessment Questionnaire
IL: Interleukin
JSN: Modified Sharp joint space narrowing
MMP3: Matrix metalloproteinase-3
mTSS: Modified total Sharp score
MTX: Methotrexate
RA: Rheumatoid arthritis
RANK: Receptor activator of nuclear factor kappa B
RANKL: Receptor activator of nuclear factor kappa B ligand
TNF-α: Tumor necrosis factor-alpha
VAS: Visual analogue scale
